# Organic-Cation Modulated Assembly Behaviors of a Ureidopyrimidone-Grafting Cluster

**DOI:** 10.3390/molecules28093677

**Published:** 2023-04-24

**Authors:** Fengrui Jiang, Jiaxu Wang, Bao Li, Lixin Wu

**Affiliations:** State Key Laboratory of Supramolecular Structure and Materials, College of Chemistry, Jilin University, Changchun 130012, China

**Keywords:** polyoxometalate, supramolecular interaction, ureidopyrimidone, organic-cation

## Abstract

Ureidopyrimidone (UPy) is an important building block for constructing functional supramolecular polymers and soft materials based on their characteristic quadruple hydrogen bonds. While the evidence from the single-crystal X-ray diffraction data for the existence of linear hydrogen bonding has still been absent up to now. To obtain the crystals of UPy-containing molecules with high quality, enhanced rigidity and crystallinity are expected. Herein, an inorganic Anderson–Evans type cluster [Mn(OH_)6_Mo_6_O_18_]^3−^, which can provide suitable stiffness and charge, is used as a linker to covalently anchor two UPy units. The prepared organic–inorganic polyanion with three negative charges has a linear architecture, which is prone to form an infinite one-dimensional structure based on the supramolecular forces. The results indicate that the combination models of UPy units can be conveniently modulated by organic counter cations with different sizes, and therefore three unreported models are observed under various conditions. The present study gives a unique understanding of the intermolecular interactions in UPy-based supramolecular polymers and also provides a simple tuning method, which benefits the construction of functional materials and the adjustment of their properties.

## 1. Introduction

The hydrogen bond is one of the important driving forces for the construction of supramolecular assemblies [1]. However, due to the relatively low bond energy of individual hydrogen bond, they generally cannot provide enough energy for building well-organized assemblies and multiple hydrogen bonds are needed to act synergistically [2,3]. Utilizing this strategy, various supramolecular assemblies based on multiple hydrogen bonds have been designed and prepared, which makes small molecules express the features of polymers, resulting in abundant functions [4,5]. Of course, it is useful and important to make the connection modes between building units clear, and therefore precisely control them for achieving designed functions, as well as a better understanding of the relationship between structures and properties [6]. While it is not easy to obtain detailed structural information in soft materials, and alternately, as a powerful tool for the analysis of the intermolecular interactions, single-crystal X-ray diffraction data can provide detailed atomic positions and their connection modes including bond lengths and bond angles [7]. The key bridge between soft materials and single crystals is to select a suitable building unit, which can express the compatible connection modes in both states.

**Scheme 1:**
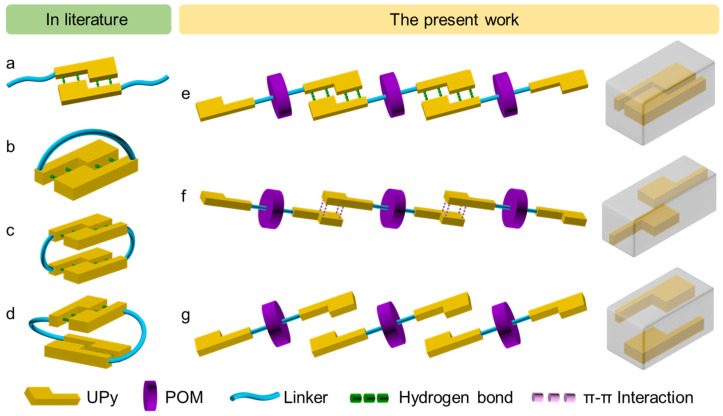
The combination models of UPy-containing molecules in crystals in the literature and this work. (**a**) Two-molecule dimer, (**b**) single-molecule dimer in a circular format, (**c**) two-molecule *syn* [2 + 2] dimer, (**d**) two-molecule *anti* [2 + 2] dimer, (**e**) 1D hydrogen-bonded structure, (**f**) 1D π-π interaction-based structure, and (**g**) discrete structure.

Ureidopyrimidone (UPy), as a typical group in which hydrogen bond donors and acceptors coexist, can form a quadruple hydrogen bonding structure through the combination of two components, thereby being used to construct stable supramolecular assemblies [8]. In the typical UPy-containing supramolecular crystals, when UPy connects to the molecule in a single-headed modification, it often forms a dimer through the quadruple hydrogen bonding between the two molecules (Scheme 1a) [9,10,11,12]. When UPy is connected to both ends of one molecule with double-headed modification, it often self-polymerizes in the form of a circular dimer (Scheme 1b) or binds in a two-molecule [2 + 2] mode with *syn* (Scheme 1c) or *anti* (Scheme 1d) conformation [13,14,15,16]. Although the UPy two-headed structure has the possibility of forming a one-dimensional (1D) chain structure through quadruple hydrogen bonding, the intermediate linking group is generally a flexible organic component, and in the reported crystals, they all dimerize in different forms. By changing the linker from a flexible organic unit to a more rigid inorganic component, it is expected to suppress the formation of dimers and obtain a 1D linear structure. In addition, different from the neutral organic linkers in most cases, the charges of the inorganic linkers are adjustable, which introduces the counter ions into the system, increasing variables for the modulation of the structures.

Polyoxometalates (POMs) are a class of clusters formed by early transition metal ions (V, Mo, W, Nb, Ta, et al.) in high-valence through the corner-, edging- or facing-shared O atoms [17,18]. They have the characteristics of rich compositions and diverse structures and have been widely used in the fields of energy, magnetism, catalysis, and materials science [19,20,21,22,23]. The bridging O atoms of some kinds of POMs can be replaced by organic molecules to obtain covalently modified organic–inorganic hybrids [24]. Among them, the method of replacing the coordinating O atoms around the central heteroatom of Anderson–Evans type POMs with triol ligands can introduce organic components with various structures and functional properties into POMs in the form of one- or two-side modification, thereby achieving synergistic functionalization of organic and inorganic components [25,26,27]. For example, by modifying two adeninyl groups on both sides of Anderson–Evans type POM and utilizing the double hydrogen bonds between two adeninyl groups, the main-chain type POM supramolecular polymer can be constructed, and exhibits thermally induced hybrid gel behaviors [28]. These results indicate that 1D linear supramolecular polymers can be constructed by modifying organic groups with suitable interaction sites on both sides of POMs by taking advantage of the rigidity.

In this work, two UPy groups are covalently and symmetrically modified on both sides of the Mn^III^-centered Anderson–Evans type POM through an amidation reaction. By regulating the counter cation of the cluster, we successfully obtain three unreported packing types of UPy-based supramolecular assembly structures, including 1D supramolecular assembly based on quadruple hydrogen bond (Scheme 1e), 1D linear structure based on π-π interaction (Scheme 1f) and discrete structure without obvious interaction (Scheme 1g). The above assembly structures show that the introduction of rigid inorganic components has an important impact on the combination mode of UPy, which not only increases the crystallinity of the hybrids but also brings counter cations into the structure and enhances the modulation ability of the system.

## 2. Results and Discussion

### 2.1. Synthesis of UPy-Containing Crystals

**Figure 1:**
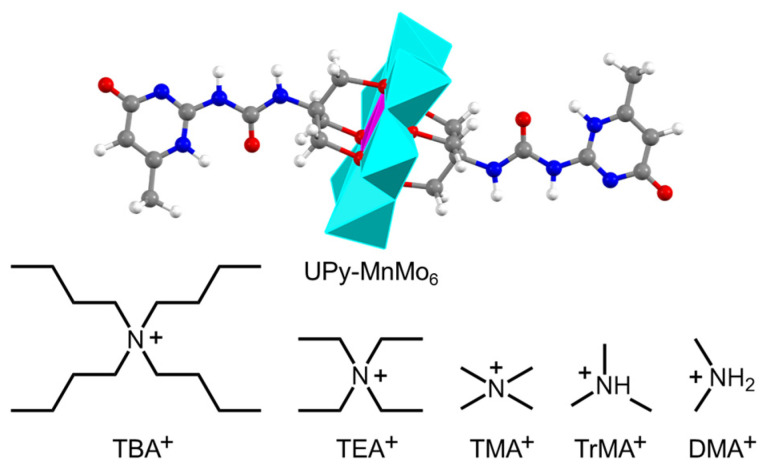
Combined ball-and-stick and polyhedron representation of polyanion UPy-MnMo_6_ (**up**) and chemical structures of five organic counter cations (**down**) in crystals. Pink octahedron: {MnO_6_}; cyan octahedra: {MoO_6_}; red ball: O; blue ball: N; gray ball: C; light ball: H.

UPy modified cluster is synthesized following a post-grafted method. (Hydroxymethyl)aminomethane modified inorganic cluster with tetrabutylammonium (TBA^+^) as counterion is firstly synthesized according to the literature (TBA-NH_2_-MnMo_6_) [29] and then N,N’-carbonyldiimidazole (CDI) is used to activate 2-amino-4-hydroxy-6-methyl pyrimidine (MIC) [30]. The resulting CDI-MIC is further used to react with TBA-NH_2_-MnMo_6_, generating [N(C_4_H_9_)_4_]_3_MnMo_6_O_18_[C_10_H_13_N_4_O_5_]_2_ (TBA-UPy-MnMo_6_). After that, the counterion exchange reaction between TBA-Upy-MnMo_6_ and excess NaClO_4_ in dimethyl sulfoxide and acetonitrile affords the product of Na_3_MnMo_6_O_18_[C_10_H_13_N_4_O_5_]_2_ (Na-UPy-MnMo_6_). The dissolved TBA-Upy-MnMo_6_ in the mixed solvents of H_2_O, acetonitrile, and dimethylformamide results in the formation of crystal **1** after a solvent-evaporation process for two days. With a similar strategy, the counter cation of Na-UPy-MnMo_6_ can be replaced by tetraethylammonium (TEA^+^), tetramethylammonium (TMA^+^), trimethylammonium (TrMA^+^) and dimethylammonium (DMA^+^) to form crystals **2–5**, respectively, which have the same anion architecture (Figure 1). The detailed synthetic procedures and characterizations of the organically grafted POM clusters are described in the supporting information (Figures S1−S6, Table S1).

### 2.2. Combination Models of UPy in Crystals

**Figure 2:**
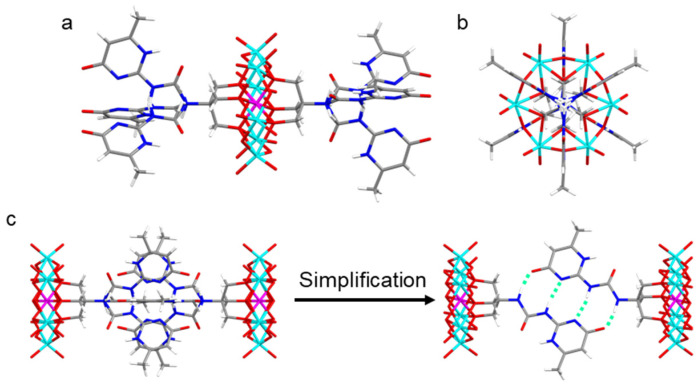
Wireframes of polyanion in crystal **1** from (**a**) the side view, and (**b**) the front view. (**c**) A simplified expression of quadruple hydrogen bonds between two adjacent polyanions in crystal **1**, and the organic group attaching on the other side of the cluster is omitted for clarity. Color code: pink for Mn, cyan for Mo, red for O, blue for N, gray for C, and white for H. The green dot lines in Figure 2c refer to the hydrogen bonds.

Although crystals **1–5** have the same polyanionic architecture, the differences between their organic counter cations influence the combination models of UPy units. When TBA^+^ is used in crystal **1**, single-crystal X-ray diffraction analysis reveals that the polyanion exhibits a classical characteristic of an Anderson–Evans type cluster with two UPy units anchoring on both sides. Crystal **1** has a space group of *R*$\overset{-}{3}$, and polyanion locates on the three-fold axis, which results in a disordered state of UPy unit with three crystallographically equivalent fragments with a dihedral angle of 120° (Figure 2a). Meanwhile, from the perpendicular view of the cluster, the disordered UPy units exhibit a hexagonal star structure with a twisted angle of 60° between each component, indicating that two UPy units are in exactly opposite directions in crystal (Figure 2b). Such arrangements of two UPy units are beneficial for the formation of the quadruple-hydrogen-bonded structure along the 1D direction. Although all the UPy parts are in the three-fold disordered state, the simplified results demonstrate that two adjacent UPy units undergo an intramolecular dimerization through a DDAA-AADD (D refers donor, and A refers acceptor) type of hydrogen-bonded array (Figure 2c), in which the distance of donor and acceptor in the outer N–H⋯O (2.603 Å, 162.42°) is a little shorter than that of the inner N–H⋯N (3.028 Å, 172.11°). In addition, the intramolecular interaction between the urea carbonyl group and N–H of the heterocyclic ring (2.491 Å, 131.48°) results in the formation of a six-membered conjugated hydrogen-bonded network, which enhances the planarity of the resulted dimer. The rigid molecular structure and strong intermolecular hydrogen bonds result in the formation of 1D infinite assemblies in the solid state. It should be noted that due to the totally disordered state of organic cations in crystals, TBA^+^ cannot be distinguished from single-crystal X-ray diffraction data. While combining the results of ^1^H NMR and element analysis, three TBA^+^ are conformed with one polyanion, which also realizes the demand of charge balance (Figure S7, Table S2).

**Figure 3:**
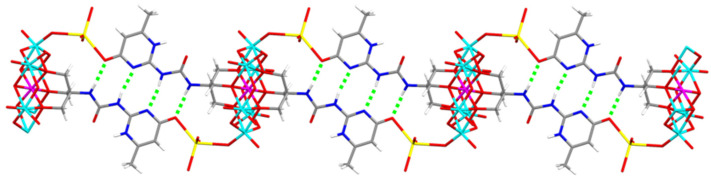
One-dimensional quadruple hydrogen bonding structure in crystal **2**. Color code: pink for Mn, cyan for Mo, yellow for Na, red for O, blue for N, gray for C, and white for H. The green dot lines refer to the hydrogen bonds.

To investigate the influence of counter cations on the packing models of UPy, TEA^+^ with a shorter alkyl chain compared with TBA^+^ is used, generating crystal **2**. As this crystal is prepared through an ion-replacement procedure from Na-UPy-MnMo_6_ in an aqueous solution, there is one Na^+^ and two TEA^+^ in the structure. The inductively coupled plasma atomic emission spectrometry (ICP-AES) result of crystal **2** also confirms that the molar ratio of Mn:Na:Mo is 1:1:6 (Table S3). Na^+^ is five-coordinated by three water O atoms, one O atom belongs to the pyrimidine ring of one cluster, and one terminal O atom sources from another cluster. The existence of Na^+^ shortens the distance of neighboring clusters, which benefits the formation of intermolecular hydrogen bonds. Similar to that in crystal **1**, a quadruple-hydrogen-bonded structure in the form of DDAA-AADD mode is also achieved in crystal **2**, in which the outer N–H⋯O (2.776 Å, 155.27°) hydrogen bond is a little stronger than the inner N–H⋯N (2.982 Å, 166.49°). In addition, the double-decorated feature of the cluster along with its rigidity results in the formation of a 1D infinite hydrogen bonding structure (Figure 3).

**Figure 4:**
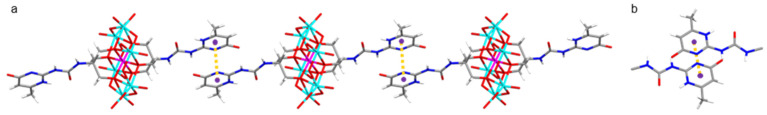
(**a**) One-dimensional π-π interaction-based structure in crystal **3**. (**b**) The distance between the adjacent rings in crystal **3**, showing a π-π interaction between them. Color code: pink for Mn, cyan for Mo, red for O, blue for N, gray for C, and white for H. The orange dot line refers to π-π interaction.

In crystals **1** and **2**, UPy units are all arranged through the intermolecular quadruple hydrogen bonding, therefore forming a 1D structure. Although counter cations influence the packing structures of crystals **1** and **2**, they do not affect the combination model of UPy. In the further study, we continue to shorten the alkyl chain length of counter cation and TMA^+^ is selected, which has only one carbon atom on each branch. As shown in Figure 4a, though a similar 1D infinite structure is also discovered in the solid state as that in crystals **1** and **2**, crystal **3** shows a totally different UPy packing model. In crystals **1** and **2**, UPy units exist in an edge-to-edge manner and therefore result in the formation of the quadruple hydrogen bonding between neighbors. While in crystal **3**, the neighboring UPy units are arranged in a face-to-face style, which restricts the hydrogen bonds between them, and instead the π-π interactions become the main driving force with a distance between the two adjacent rings of 3.57 Å (Figure 4b). The present combining manner of UPy has not been observed in other systems, in which hydrogen bonds are compressed by π-π interactions. The two hydrogen bond donors of the UPy unit do not form hydrogen bonds with the hydrogen bond acceptors of another UPy unit as that in crystals **1** and **2**, and instead of forming hydrogen bonds with inorganic clusters or lattice water molecules. As this type of combination manner of UPy units can form similar extended structures to those based on hydrogen bonding, it is hard to be distinguished through other characterization methods except single-crystal X-ray diffraction.

**Figure 5:**
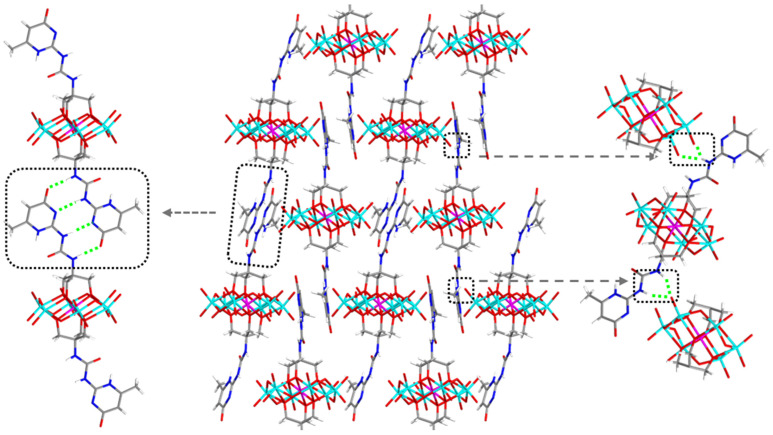
The first type of polyanion in crystal **4** shows a quadruple-hydrogen-bonded structure formed between the adjacent UPy units (**left**), and the second type of polyanion in crystal **4** (**right**), shows the hydrogen bonds of UPy units with the adjacent inorganic clusters. Color code: pink for Mn, cyan for Mo, red for O, blue for N, gray for C, and white for H. The green dot lines refer to the hydrogen bonds.

In a further study, in the presence of the same polyanion, one methyl of TMA^+^ is replaced by a proton, generating crystal **4**. Though the counter cation in crystal **4** has a similar architecture to that in crystal **3**, it still expresses a reduced size. Single-crystal X-ray diffraction results indicate that two crystallographically independent polyanions exist in the asymmetric unit of crystal **4**, which express two different combination styles of UPy units. For the first type of polyanions, classical quadruple hydrogen bonding is formed as those in crystals **1** and **2**, resulting in the formation of a 1D supramolecular chain (Figure 5). In the formed DDAA-AADD array in crystal, the outer N–H⋯O (2.854 Å, 165.04°) is also shorter than the inner N–H⋯N (2.991 Å, 169.13°), similar to those in crystals **1** and **2**. For the second type of polyanions, UPy units do not interact with neighboring ones in the edge-to-edge or face-to-face style, which prevents the formation of hydrogen bonds or π-π interactions. Instead, as the hydrogen bonding donors, UPy units form two N–H⋯O hydrogen bonds (2.951 Å, 150.75° and 3.005 Å, 155.64°) with the same hydrogen bonding acceptor sourcing from the neighboring inorganic cluster (Figure 5). In this case, there is no obvious interaction between UPy units, which has not been reported up to now. When two methyls of TMA^+^ are replaced by two protons, crystal **5** is obtained, which shows a similar UPy combination model to that in crystal **4** (Figure S8).

### 2.3. Organic-Cation Modulated Combination Model of UPy in Crystals

**Figure 6:**
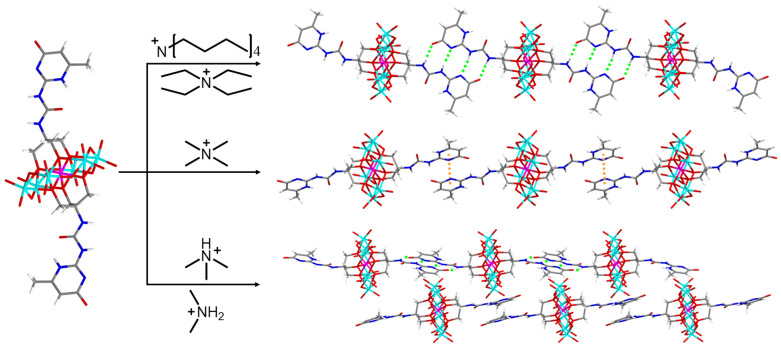
The accumulation behaviors of UPy-MnMo_6_ modulated by different organic cations. Color code: pink for Mn, cyan for Mo, red for O, blue for N, gray for C, and white for H. The green dot lines refer to the hydrogen bonds. The orange dot lines refer to the π-π interaction.

As discussed above, by changing counter cations of polyanion, the UPy units express different existing manners, from quadruple hydrogen bonding, π-π interactions to no obvious interaction (Figure 6). As all these crystals have the same polyanion, and are synthesized under similar conditions, and therefore organic cations become the main factor for the modulation of UPy combination manner. For organic cations used in this study, TBA^+^, TEA^+^, and TMA^+^ all have the same tetrahedron architecture with an “N” as core and four arms with 4, 2, and 1 carbon atoms, respectively. For TrMA^+^- and DMA^+^-containing structures, their counter cations can also be seen as the same architecture as that of TMA^+^, except that the one and two methyls are replaced by hydrogen atoms. All the features of the counter cations make the size become the main factor for the modulation of UPy packing.

**Figure 7:**
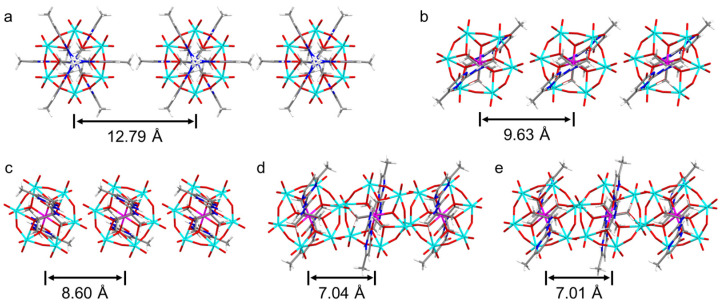
The longitudinal distance between adjacent clusters of crystal (**a**) **1**, (**b**) **2**, (**c**) **3**, (**d**) **4**, and (**e**) **5** modulated by the organic cations with different sizes. Color code: pink for Mn, cyan for Mo, red for O, blue for N, gray for C, and white for H.

In the presence of quadruple hydrogen bonding or π-π interactions, all inorganic polyanions in five crystals assemble into 1D infinite structures and organic cations located in the space between chains. When the 1D chains are observed along the symmetry axis of an inorganic cluster, the longitudinal distance between two adjacent clusters can be easily calculated. From crystals **1** to **5**, this distance successively decreases accompanied by the size descent of counterions (Figure 7), which is caused by the more compact stacking of clusters with smaller counterions. During the formation process of crystals, molecules tend to stack in a most compact mode influenced by lattice energy. When the 1D infinite assemblies constructed by the quadruple-hydrogen-bonded interaction of UPys start to stack, TBA^+^ and TEA^+^ with bigger molecular volumes are large enough to fill the void of the stacked 1D structure. While the volume of TMA^+^ cannot cover the void even with the 1D assemblies stacking in the most compact mode. Hence, lattice energy dominates the packing pattern of TMA^+^ and polyanion rather than quadruple hydrogen bonding interaction, resulting in the formation of interlaced and compact 1D structure based on π-π interaction. Analogously, balanced by the lattice energy and quadruple hydrogen bonding interaction, only half of the Upys participate in the formation of a dimeric array for crystals **4** and **5**.

### 2.4. The Influence of Combination Models of UPy on the Thermal Stability and FT-IR Spectra

**Figure 8:**
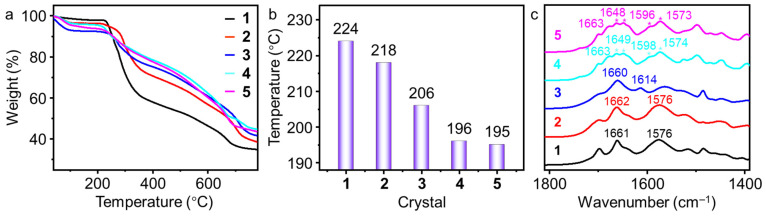
(**a**) TGA curves of crystals **1**–**5** and (**b**) the column diagram of their decomposition temperatures. (**c**) FT-IR spectra of crystals **1**–**5**, showing the different combination models.

The counter cations with different sizes dominate the packing of UPy units, resulting in various combination models. The changes in intermolecular interaction between UPy units in five crystals also influence their thermal stability, which is proved by thermogravimetric analysis (TGA). As shown in Figure 8a, all five crystals maintain the integrated structure below 190 °C except for the loss of crystallization water molecules. The electrostatic attraction between organic cations and polyanions, quadruple hydrogen bonding or π-π interaction existing in the adjacent polyanions are responsible for the relatively high thermal stability of these crystals. It is interesting that when full quadruple hydrogen bonds exist in the crystals, their decomposition temperature can reach up to 224 and 218 °C for crystals **1** and **2** (Figure 8b). As a comparison, for crystals **4** and **5**, only half of the polyanions are linked together through quadruple hydrogen bonds, and they show a relatively lower decomposition point of 196 and 195 °C, respectively. The decreased decomposition temperatures indicate that hydrogen bonds seem to play an important role for the stabilization of crystals. For crystal **3**, due to the existence of π-π interaction, it expresses a moderate decomposition temperature of 206 °C. The thermal stability behaviors of crystals **1–5** show a slow decrease with the changes in intermolecular interactions, in which strong binding generates higher stability and weak binding results in lower stability.

FT-IR spectra are also used to identify the supramolecular interactions in crystals **1–5**. As shown in Figure 8c, the peak positions of the urea for five crystals are very close and all concentrate at ca. 1662 cm^−1^. The FT-IR spectrum of crystal **3** shows a broadened stretching band at 1614 cm^−1^ originating from the pyrimidine ring, this vibration band clearly shifts to a lower wavenumber of 1576 cm^−1^ for crystals **1** and **2**, further indicating the formation of a quadruple-hydrogen-bonded structure. Due to half polyanion being involved in the formation of quadruple hydrogen bonds for crystals **4** and **5**, the band appearing at ca. 1573 cm^−1^ belongs to the pyrimidine ring with intermolecular hydrogen-bonded interaction. Influenced by the interaction between urea and terminal oxygen of cluster, the remanent pyrimidine ring without formation of intermolecular hydrogen bond appears at ca. 1597 cm^−1^. The same influence can also be observed for urea, except for the peak at 1663 cm^−1^, half polyanion with non-involved in the formation of quadruple hydrogen bond emerges at ca. 1648 cm^−1^. FT-IR results provide powerful evidence of UPys’ arrangement manners in five crystals.

### 2.5. Assembled Behaviors in Aqueous Solution

**Figure 9:**
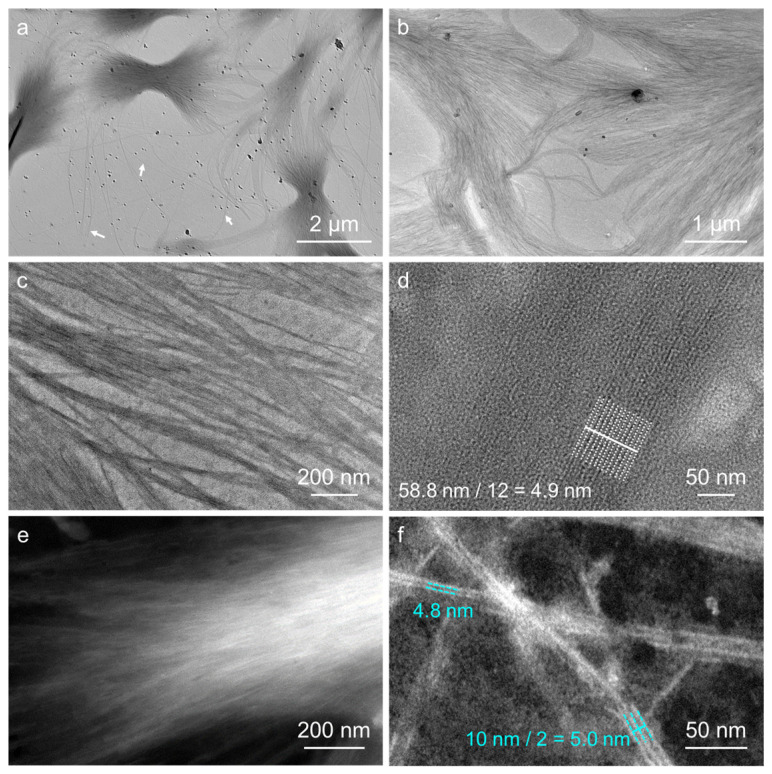
(**a**–**d**) TEM images of fibrous assemblies of Na-UPy-MnMo_6_ with different magnifications. (**e**,**f**) HAADF-TEM images of fibrous assemblies of Na-UPy-MnMo_6_. White arrows refer single fibers.

Although the 1D supramolecular polymer based on UPy units can be constructed in crystals, the tough and rigid structure of crystals makes them unsuitable for further processing. Therefore, the self-assembly of UPy in solution is helpful for the further development of the modified clusters’ functionalization. In order to verify the capability of UPy-MnMo_6_ to form 1D infinite assemblies based on quadruple hydrogen bonds in an aqueous solution, UPy-containing polyanion with Na^+^ as counter cation is selected as the building block. For aqueous solutions of Na-UPy-MnMo_6_ with different concentrations from 1 to 20 mg mL^−1^, after incubating the solution for 3 h at room temperature, the dynamic light scattering (DLS) curve shows the maximum hydrodynamic diameter of formed assemblies increases with the rise of concentration, indicating the formation of assemblies in a higher concentration (Figure S9). When the concentration of Na-UPy-MnMo_6_ is lower than 2 mg mL^−1^ (Figure S10a,b), no ordered assemblies are observed from transmission electron microscopic (TEM). While the organized and disorganized assemblies emerge together with a concentration of 3 mg mL^−1^ (Figure S10c), indicative of the concentration dependence of Na-UPy-MnMo_6_ during the assembly process. When the concentration of Na-UPy-MnMo_6_ is higher than 4 mg/mL, only entangled fibrous assemblies are detected from TEM images (Figure S10d–f), which demonstrates the consistency of monomer packing mode in a high concentration. After that, the inner structure of Na-UPy-MnMo_6_-based fibers is probed with a concentration of 5 mg mL^−1^ (Figure 9a–c), and the large size of successively fibrous assemblies in several micrometers with an average separation distance of about 4.9 nm are clearly observed (Figure 9d). High-angle annular dark-field scanning TEM (HAADF-STEM) also demonstrates the distance between two adjacent fibers is about 4.9 nm (Figure 9e,f). The theoretical length of UPy-containing polyanion is about 1.6 nm and the diameter of the disk-like inorganic cluster is 0.9 nm [31], thus the distance of 4.9 nm cannot be the space between two clusters in the single theoretical 1D assemblies formed by a quadruple hydrogen bond. The most possible reason should be a few single 1D assemblies interacting with each other forming the entangled fibers with a diameter of about 4.9 nm, the entangled fibers are in a metastable state, and further stacking leads to the evolution of fibrous assemblies with a larger size.

## 3. Materials and Methods

### 3.1. General Methods and Materials

^1^H NMR spectra were recorded on a Bruker AVANCE 500 MHz spectrometer (Bremen, Germany) by using tetramethylsilane (TMS) as an internal reference. The Electrospray Ionization Mass Spectrometry (ESI-MS) was performed on an Agilent1290-micrOTOF Q II spectrometer. Organic elemental analysis (EA) was carried out on a Vario micro cube from Elementar, Langenselbold, Germany. Transmission electronic microscopic (TEM) and high-angle annular dark-field scanning TEM (HAADF-STEM) were conducted on a JEOL JEM-2100F under an accelerating voltage of 200 kV without staining. ICP-AES was carried out on the Micromeritics ASAP 2020 Plus (PerkinElmer, Waltham, MA, USA). Fourier transform infrared spectra (FT-IR) was collected on a Bruker Vertex 80 V spectrometer (Bremen, Germany) equipped with a DTGS detector (32 scans) in a resolution of 4 cm^−1^ in pressed KBr pellets. Thermal stability of compounds was evaluated by a Q500 Thermal Analyzer (New Castle TA Instruments, New Castle, DE, USA) with a flowing N_2_ atmosphere and a heating rate of 10 °C/min single-crystal X-ray diffraction indexing, and data collection was performed on a Bruker D8 Venture diffractometer (Bruker, Bremen, Germany) with graphite-monochromated Mo Kα (λ = 0.71073 Å) at 293 K. All crystal structures were solved using SHELXTT and refined by full-matrix least-squares fitting for F^2^ via the Olex^2^ software. The SQUEEZE function in the PLATON program of the SHELXTL software was used to remove the residual electron density, which could not be modeled precisely. All non-H atoms were refined with anisotropic thermal parameters. A summary of the crystallographic data and structural refinements for crystals **1**–**5** is listed in Table 1.

### 3.2. Synthesis of Five Crystals

Synthesis of [N(C_4_H_9_)_4_]_3_MnMo_6_O_18_[C_10_H_13_N_4_O_5_]_2_·6(H_2_O) (1). The pure TBA-UPy-MnMo_6_ (100.0 mg, 0.046 mmol) was dissolved in the mixed solvents (1 mL DMF, 14 mL H_2_O, 10 mL CH_3_CN) at room temperature. With solvent evaporation for about two days, orange crystals were obtained (69.3 mg, yield 69.3%). ^1^H NMR (500 MHz, DMSO-d_6_, δ = ppm): 64.36 (br, 12H), 11.21 (s, 2H), 9.51 (s, 2H), 8.61–7.87 (br, 2H), 6.46 (s, 4H), 5.78 (s, 2H), 3.17 (t, 24H), 2.12 (s, 6H),1.57 (m, 24H), 1.32 (m, 24H), 0.94 (t, 36H). Elemental Analysis: (Calculated: C: 37.39%, H: 6.18%, N: 7.05%, Found: C: 37.25%, H: 6.26%, N: 6.87%).

Synthesis of Na[N(C_2_H_5_)_4_]_2_MnMo_6_O_18_[C_10_H_13_N_4_O_5_]_2_·12H_2_O (2). A mixture of Na-UPy-MnMo_6_ (50.0 mg, 0.033 mmol) and TEA·Br (30.9 mg, 0.15 mmol) was firstly dissolved in 10 mL deionized water, the resultant solution was allowed to stay at room temperature for 2 days. The products deposited from the solution as orange crystals were obtained by filtration (47.5 mg, yield: 74.1%). ^1^H NMR (500 MHz, DMSO-*d_6_*_,_ δ = ppm): 64.38 (br, 12H), 11.54–10.56 (br, 2H), 10.20–8.97 (br, 2H), 8.72–7.70 (br, 2H), 5.82 (s, 2H), 3.20 (q, 16H), 2.15 (s, 6H), 1.18 (t, 24H). Elemental Analysis: (Calculated: C: 22.10%, H: 4.64%, N: 7.16%, Na: 1.17%, Mn: 2.81%, Mo: 29.42%, Found: C: 22.24%, H: 4.82%, N: 7.30%, Na: 1.08%, Mn: 2.69%, Mo: 29.41%).

Synthesis of H[N(CH_3_)_4_]_2_MnMo_6_O_18_[C_10_H_13_N_4_O_5_]_2_·3H_2_O (3). A mixture of Na-UPy-MnMo_6_ (50.0 mg, 0.033 mmol) and TMA·Cl (16.4 mg, 0.15 mmol) was firstly dissolved in 10 mL deionized water, the resultant solution was allowed to stay at room temperature for 2 days. The products deposited from the solution as orange crystals were obtained by filtration (47.5 mg, yield: 62.1%). ^1^H NMR (500 MHz, DMSO-*d_6_*_,_ δ = ppm): 64.38 (br, 12H), 11.54–10.56 (br, 2H), 10.05–9.03 (br, 2H), 8.72–7.74 (br, 2H), 5.82 (s, 2H), 3.10 (s, 24H), 2.15 (s, 6H). Elemental Analysis: (Calculated: C: 20.25%, H: 3.46%, N: 8.44%, Mn: 3.30%, Mo: 34.67%, Found: C: 20.24%, H 3.33%, N: 8.47%, Mn: 3.18%, Mo: 34.11%).

Synthesis of H_1.5_[NH(CH_3_)_3_]_1.5_MnMo_6_O_18_[C_10_H_13_N_4_O_5_]_2_·4H_2_O (4). A mixture of Na-UPy-MnMo_6_ (100.0 mg, 0.066 mmol) and TrMA·Cl (28.7 mg, 0.30 mmol) was firstly dissolved in 10 mL deionized water, the resultant solution was allowed to stay at room temperature for 5 days. The products deposited from the solution as orange crystals were obtained by filtration (41.4 mg, yield: 40.1%). ^1^H NMR (500 MHz, DMSO-*d_6_*_,_ δ = ppm): 64.38 (br, 12H), 11.74–10.79 (br, 2H), 9.95–9.12 (br, 2H), 8.66–7.96 (br, 2H), 5.81 (s, 2H), 2.79 (q, 13.5H), 2.13 (s, 6H). Elemental Analysis: (Calculated: C: 18.16%, H: 3.14%, N: 8.21%, Mn: 3.39%, Mo: 35.52%, Found: C: 18.36%, H: 3.21%, N: 8.26%, Mn: 3.29%, Mo: 34.58%).

Synthesis of H_1.5_[NH_2_(CH_3_)_2_]_1.5_MnMo_6_O_18_[C_10_H_13_N_4_O_5_]_2_·8H_2_O (5). A mixture of Na-UPy-MnMo_6_ (100.0 mg, 0.066 mmol) and DMA·Cl (24.5 mg, 0.30 mmol) was firstly dissolved in 10 mL deionized water, the resultant solution was allowed to stay at room temperature for 5 days. The products deposited from the solution as orange crystals were obtained by filtration (46.0 mg, yield: 45.3%). ^1^H NMR (500 MHz, DMSO-*d_6_*_,_ δ = ppm): 64.36 (br, 12H), 11.45–10.86 (br, 2H), 9.70–9.18 (br, 2H), 8.45–7.78 (br, 2H), 5.78 (s, 2H), 2.55 (s, 9H), 2.12 (s, 6H). Elemental Analysis: (Calculated: C: 16.52%, H: 3.35%, N: 7.96%, Mn: 3.29%, Mo: 34.43%, Found: C: 16.66%, H: 3.47%, N: 7.98%, Mn: 3.22%, Mo: 34.07%).

## 4. Conclusions

By covalently modifying UPy units on both sides of the Mn-centered Anderson–Evans type cluster, five crystals with the same polyanion and different counter cations are synthesized. The change of counter cations has significant influences on the combination models of UPy units, resulting in three unreported styles in crystals, which is similar with the process of anion-assisted supramolecular polymerization [32]. When TBA^+^ and TEA^+^ are used to balance the charge of polyanion, their relatively large molecular sizes promote the formation of quadruple hydrogen bonds between adjacent UPy units to achieve a 1D chain structure. With TMA^+^ as a counter cation, π-π interactions are found between the rings of UPy, which have never been reported in the literature. For TrMA^+^ and DMA^+^ as counter cations, only half of the UPy-containing clusters are involved to form quadruple hydrogen bonds, while the other half of clusters show no obvious interaction between the adjacent UPy units. The counter actions are considered to be the main factor for the modulation of UPy combination manners through their distribution between the 1D chains of clusters in various molecular sizes. In addition, when Na^+^ is applied as a counter cation, a fibrous structure can be achieved, indicative of the similar combination model of UPy units in solution and in the solid state. The research presented here gives clear evidence for the combination models of UPy units in crystals and also proves their existence in the solution, which helps understand the assembly mechanism of UPy-containing compounds.

## Figures and Tables

**Table 1:** The summary of crystal data and structural refinements for crystals **1**–**5**.

Item	1	2	3	4	5
Formula	[N(C_4_H_9_)_4_]_3_MnMo_6_O_18_[ C_10_H_13_N_4_O_5_]_2_·6(H_2_O)	Na[N(C_2_H_5_)_4_]_2_MnMo_6_O_18_[ C_10_H_13_N_4_O_5_]_2_·12H_2_O	H[N(CH_3_)_4_]_2_MnMo_6_O_18_[ C_10_H_13_N_4_O_5_]_2_·3H_2_O	H_1.5_[NH(CH_3_)_3_]_1__._ _5_MnMo_6_O_18_[C_10_H_13_N_4_O_5_]_2_· 4H_2_O	H_1.5_[NH_2_(CH_3_)_2_]_1.5_MnMo_6_O_18_[C_10_H_13_N_4_O_5_]_2_·8H_2_O
F.W. (g mol^−1^)	2292.53	1956.74	1660.41	1620.82	1671.85
Crystal system	Trigonal	Triclinic	Monoclinic	Triclinic	Triclinic
S.G.	*R* $\overset{-}{3}$	*P* $\overset{-}{1}$	*P*2_1_/*n*	*P* $\overset{-}{1}$	*P* $\overset{-}{1}$
a (Å)	22.1032 (5)	12.3293 (9)	14.112 (3)	13.6400 (6)	13.7866 (5)
b (Å)	22.1032 (5)	12.9719 (8)	10.8510 (16)	14.0770 (7)	14.0074 (4)
c (Å)	16.4882 (5)	13.2711 (9)	18.445 (3)	16.4981 (8)	16.4860 (6)
α (deg)	90	111.614 (2)	90	94.258 (2)	80.2238 (13)
β (deg)	90	114.857 (3)	91.607 (8)	95.937 (2)	82.9721 (14)
γ (deg)	120	99.082 (3)	90	118.7227 (16)	61.3974 (11)
V (Å^3^)	6976.1 (4)	1662.9	2823.4 (8)	2735.1 (2)	2751.46 (16)
Z	3	1	2	2	2
D_c_ (g cm^−3^)	1.637	1.954	1.953	1.968	2.018
*F* (000)	3540	984	1640	1594	1650
Reflections coll./unique	36,466/3561	60,282/5850	119,356/6468	153,767/12,578	137,337/12,639
R_int_	0.0643	0.1356	0.0501	0.0731	0.1376
GOOF on *F*^2^	1.108	1.037	1.087	1.077	1.027
^a^ R_1_ [I > 2σ(I)]	0.0745	0.0546	0.0302	0.0369	0.0504
^b^ *w*R_2_ ^b^ (all data)	0.2736	0.1521	0.0994	0.1091	0.1312
CCDC no.	2,249,014	2,249,015	2,249,016	2,249,017	2,249,018

^a^ R_1_ = Σ||F_o_| − |F_c_||/Σ|F_o_|. ^b^ *w*R_2_ = Σ[*w*(F_o_^2^ − F_c_^2^)_2_]/Σ[*w*(F_o_^2^)^2^]^1/2^.

## Data Availability

Crystallographic data can be obtained free of charge from the Cambridge Crystallographic Data Centre via www.ccdc.cam.ac.uk/data_request/cif, accessed on 15 March 2023.

## Supplementary Materials

The following supporting information can be downloaded at: https://www.mdpi.com/article/10.3390/molecules28093677/s1, Figure S1: Molecular design and synthetic routes of CDI-MIC; Figure S2: Molecular design and synthetic routes of Upy-MnMo_6_ with different counterions; Figure S3: ^1^H NMR spectrum of TBA-UPy-MnMo_6_ in DMSO-*d_6_*; Figure S4: ^1^H NMR spectrum of Na-UPy-MnMo_6_ in DMSO-*d_6_*; Figure S5: ESI-MS spectrum of TBA-UPy-MnMo_6_; Table S1: Detailed assignment for the ESI-MS of TBA-UPy-MnMo_6_; Figure S6: ESI-MS spectrum of Na-UPy-MnMo_6_; Figure S7: ^1^H NMR spectrum of crystal **1** in DMSO-*d_6_*; Table S2: Organic EA results of crystal **1**; Table S3: ICP results of crystal **2**; Figure S8: The integral structure of crystal **5**; Figure S9: DLS data of Na-UPy-MnMo_6_ aqueous solution with different concentration after incubation for 3 h. Figure S10: TEM images of Na-UPy-MnMo_6_ self-assemblies in aqueous solution with different concentrations after incubation for 3 h and air-drying for 1 h [33].

## Sample Availability

Samples of the compounds are available from the authors.
