# Supplementary material for: Organic-Cation Modulated Assembly Behaviors of a Ureidopyrimidone-Grafting Cluster
Source: Molecules. 2023 Apr 24;28(9):3677. doi: 10.3390/molecules28093677 (PMC10180284; doi:10.3390/molecules28093677)
Supplement: Supplementary file 1 [file molecules-28-03677-s001.zip › molecules-2320878-supplementary.pdf]

Supplementary Information

Organic-Cation Modulated Assembly Behaviors of A Ureidopyrimidone-Grafting Cluster

Fengrui Jiang, Jiaxu Wang, Bao Li* and Lixin Wu

State Key Laboratory of Supramolecular Structure and Materials, College of Chemistry, Jilin University, Changchun 130012, P. R. China.

**E-mail: libao@jlu.edu.cn*

Contents

S1. Materials…..…………………………………………………….2

S2. Synthesis and characterizations of UPy-$MnMo_6$……….……….………2

S3. Structural characterizations of fibrous assemblies…….….…………5

S4. Structural analysis of crystals……….........................……..…...7

S5. References………………………………………...…..……...7

S1. Materials

2-amino-4-hydroxy-6-methyl pyrimidine (MIC) and *N*,*N'*-carbonyldiimidazole (CDI) were purchased from Energy Chemical. Tris(hydroxymethyl)aminomethane (NH_2-Tris) was the product of Sinopharm Chemical Reagent Co., Ltd. (SCRC). Tetrabutylammonium bromide (TBA·Br), tetraethylammonium bromide (TEA·Br), tetramethylammonium chloride (TMA·Cl), Trimethylammonium chloride (TrMA·Cl), and dimethylammonium chloride (DMA·Cl) were purchased from Aladdin. DMSO-d_6, chloroform-*d* and D_2O were purchased from Sigma-Aldrich or Cambridge Isotope Laboratories, Inc. (CIL). The organic solvents were purchased from Beijing Chemical Reagent Company. All the chemicals above were used as received. Doubly distilled water used in the experiments was homemade in the laboratory.

S2. Synthesis and characterizations of Upy-MnMo$_6$

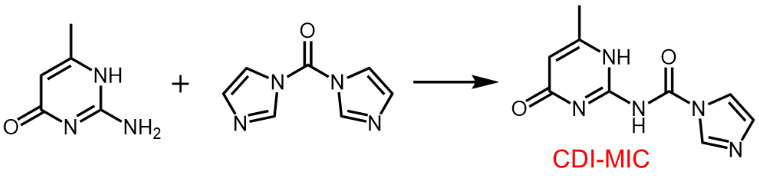

Figure S1 Molecular design and synthetic routes of CDI-MIC.

N-(6-Methyl-4-oxo-1,4-dihydropyrimidin-2-yl)-1H-imidazole-1-carboxamide (CDI-MIC). CDI-MIC was synthesized according to literatures [1]. MIC (8.0 g, 64 mmol) and CDI (14.5 g, 89.5 mmol) were suspended in DMSO (400 mL) and stirred for 2 h at 80℃.The reaction mixture was cooled down, and the resulting precipitate was filtered and washed with ethanol to yield CDI-MIC. The intermediate CDI-MIC was obtained as a white solid and dried in vacuo at 30℃ for 12 h. (12.64 g, yield 90.2%). Due to the extremely low solubility of CDI-MIC in most solvents, this compound is very hard to be characterized. Elemental Analysis: (Calculated: C: 49.80%, H: 4.38%, N: 33.19%, Found: C: 49.87%, H: 4.23%, N: 33.11%). IR: ν = 3175, 3085, 1706, 1653, 1605, 1478, 1378, 1333, 1320, 1275, 1232, 1190, 1168, 983, 853 cm^{-1}.

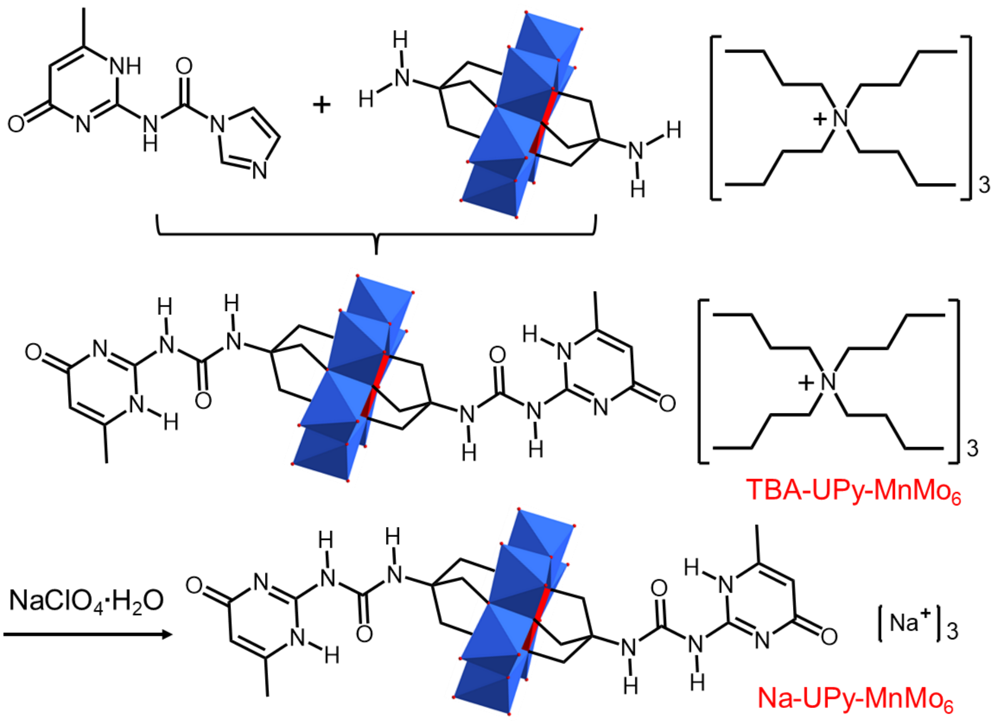

Figure S2 Molecular design and synthetic routes of UPy-MnMo$_6$ with different counterions.

Synthesis of [N(C_4H_9)$_4$]$_3$MnMo$_6$O$_{18}$[$C_{10}H_{13}N_4O_5$]$_2$ (TBA-UPy-MnMo$_6$). The starting materials of [N(C_4H_9)$_4$]$_3$Mo$_6$O$_{18}$[$C_4H_8NO_3$]$_2$ (TBA-NH$_2$-MnMo$_6$) was synthesized according to literatures [2,3]. A mixture of TBA-NH$_2$-MnMo$_6$ (1.88 g, 1 mmol) and CDI-MIC (0.48 g, 2.28 mmol) in 30 mL anhydrous DMF was stirred at room temperature for 3 h. The remnant white precipitate was removed by filtration, and then the filtrate was poured into 100 mL of diethyl ether. The resulting precipitate was collected by filtration and washed by ethanol for three times, drying to get target compound TBA-UPy-MnMo$_6$ (1.89 g, yield 86.7%). ^{1}H NMR (500 MHz, DMSO-d$_6$, δ = ppm): 64.38 (br, 12H), 11.20 (s, 2H), 9.51 (s, 2H), 8.62–7.88 (br, 2H), 6.46 (s, 4H), 5.77 (s, 2H), 3.17 (t, 24H), 2.12 (s, 6H),1.57 (m, 24H), 1.32 (m, 24H), 0.94 (t, 36H). Elemental Analysis: (Calculated: C: 37.39%, H: 6.18%, N: 7.05%, Found: C: 36.95%, H: 5.99%, N: 6.81%).

Synthesis of Na$_3$MnMo$_6$O$_{18}$[$C_{10}H_{13}N_4O_5$]$_2$ (Na-UPy-MnMo$_6$). TBA-UPy-MnMo$_6$ (1.09 g, 0.5 mmol) was dissolved in the mixed solvents of 20 ml DMSO and 30 ml CH_3CN, and then acetonitrile solution (10 mL) of sodium perchlorate monohydrate (1.12 g, 8.0 mmol) was added dropwise and stirred vigorously for 12 h at room temperature. The formed solid was collected by filtration and washed with acetonitrile for three times, drying to get target compound Na-UPy-MnMo$_6$. (683 mg, yield 89.6%). ^{1}H NMR (500 MHz, DMSO-*d*$_{6,}$ δ = ppm): 64.38 (br, 12H), 11.22 (s, 2H), 9.54 (s, 2H), 8.61–7.84 (br, 2H), 5.79 (s, 2H), 2.13 (s, 6H). Elemental Analysis: (Calculated: C: 15.74%, H: 1.72%, N: 7.34%, Na: 4.51%, Mn: 3.60%, Mo: 37.72%, Found: C: 15.44%, H: 1.62%, N: 7.50%, Na: 4.28%, Mn: 3.39%, Mo: 37.41%).

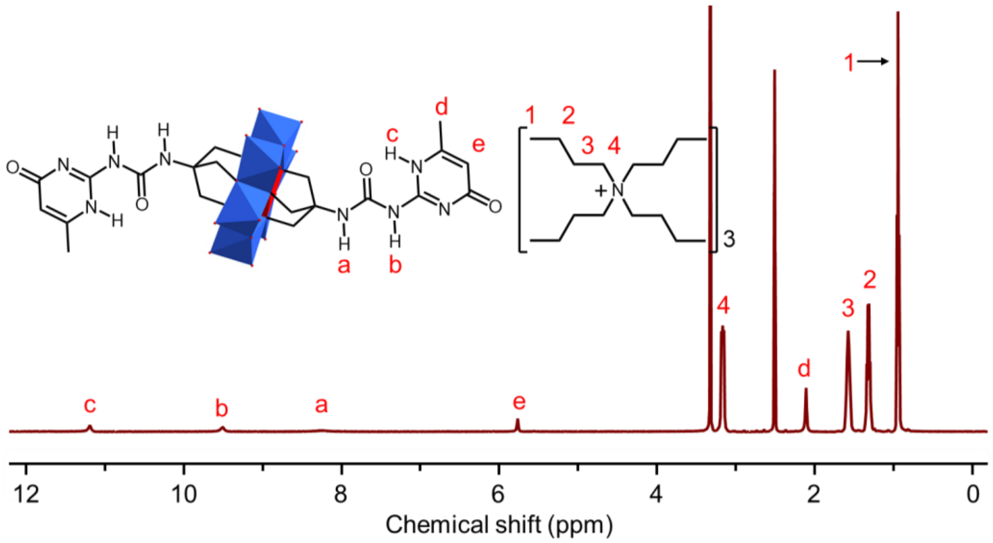

Figure S3 ^{1}H NMR spectrum of TBA-UPy-$MnMo_6$ in DMSO-d_6.

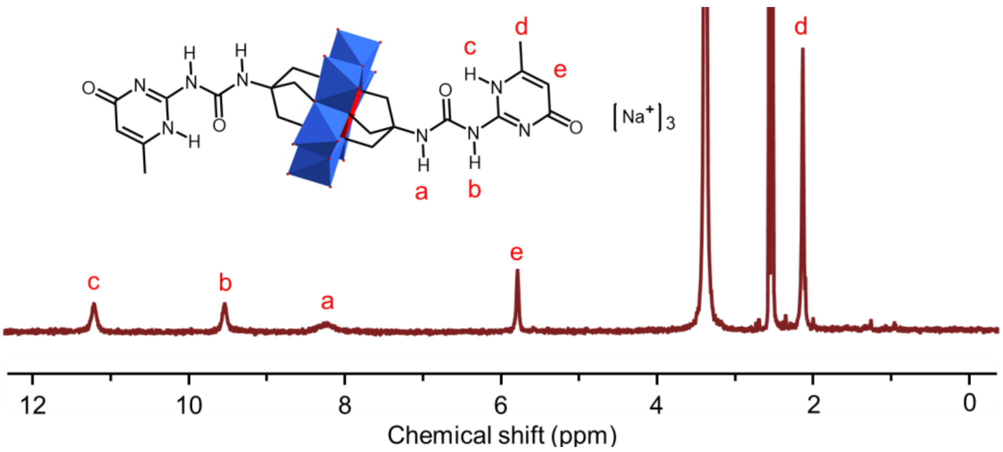

Figure S4 ^{1}H NMR spectrum of Na-UPy-$MnMo_6$ in DMSO-d_6.

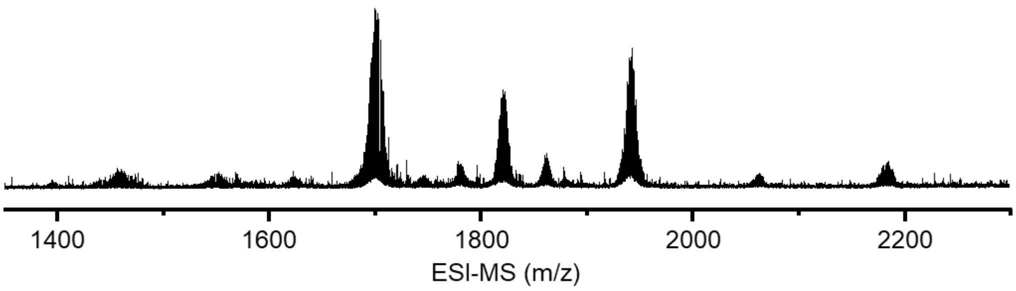

Figure S5 ESI-MS spectrum of TBA-UPy-$MnMo_6$.

Table S1 Detailed assignment for the ESI-MS of TBA-UPy-MnMo$_6$.

Chemical Formula	Charge	MW* Calculated	MW Found
$[N(C_4H_9)_4]_3MnMo_6O_{18}[C_{10}H_{13}N_4O_5]_2$-H	$1e^{-}$	2183.4	2183.3
$[N(C_4H_9)_4]_2[MnMo_6O_{18}[C_{10}H_{13}N_4O_5]_2]$	$1e^{-}$	1941.9	1942.0
$[N(C_4H_9)_4]_3[MnMo_6O_{18}[C_{10}H_{13}N_4O_5]_2]_2H$	$2e^{-}$	1821.2	1820.9
$[N(C_4H_9)_4]_1[MnMo_6O_{18}[C_{10}H_{13}N_4O_5]_2]H$	$1e^{-}$	1700.5	1700.7
$[MnMo_6O_{18}[C_{10}H_{13}N_4O_5]_2]H_2$	$1e^{-}$	1459.1	1459.5

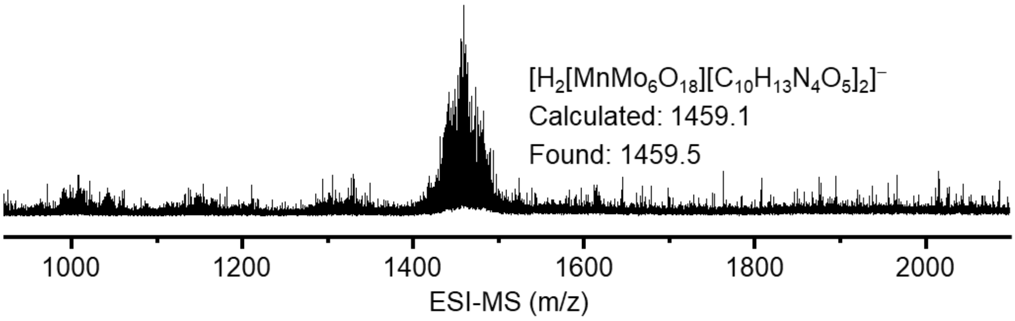

Figure S6 ESI-MS spectrum of Na-UPy-MnMo$_6$.

S3. Structural analysis of crystals

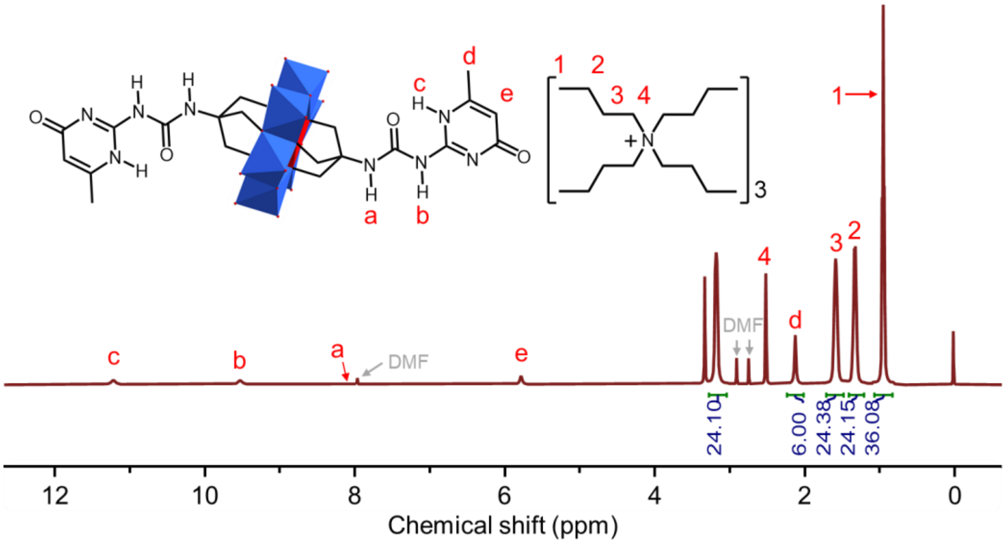

Figure S7 ^{1}H NMR spectrum of crystal **1** in DMSO-d_6.

Table S2 organic EA results of crystal **1** (unit: percentage (%)).

Elements	C	H	N
Found	37.39	6.18	7.05
Calculated	37.25	6.26	6.87
Deviation	0.14	-0.08	0.18

Table S3 ICP results of crystal **2**. ICP samples preparation process: 20 mg sample was accurately weighed and dissolved in 10 mL water, and then the solution was transferred to a 25mL volumetric flask and brought to volume by laboratory-pure water.

Elements	Na	Mn	Mo
Concentration (mg/L)	9.31	21.35	227.95
Molar weight (mmol)	0.01012	0.0097	0.0594
Molar ratio of Na:Mn:Mo	1:0.96:5.87		

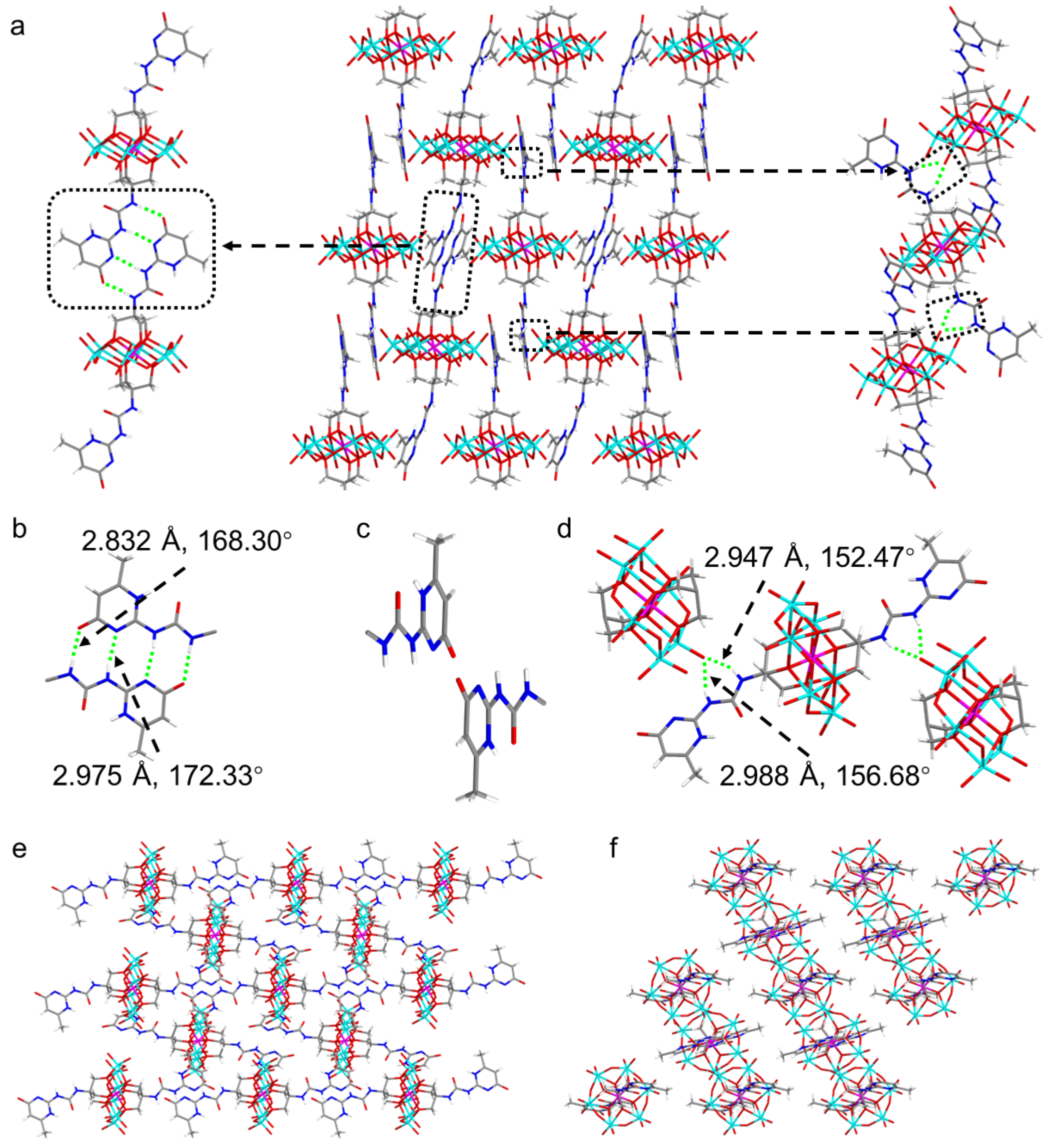

Figure S8 (a) The integral structure of crystal **5** observed from *a* axis and partial enlarged drawings. (b) The quadruple hydrogen-bonded structure between two adjacent UPys. (c) The two adjacent UPys without π-π interaction or intermolecular dimerization interaction. (d). the double intermolecular hydrogen bonding between terminal oxygen of inorganic cluster and N−H functional groups of urea. The integral structure of crystal **5** observed from *b* axis (e) and *c* axis (f). Green dotted lines represent the hydrogen bond. All solvents and DMA^+ are omitted for clarity.

S4. Structural analysis of fibrous assemblies

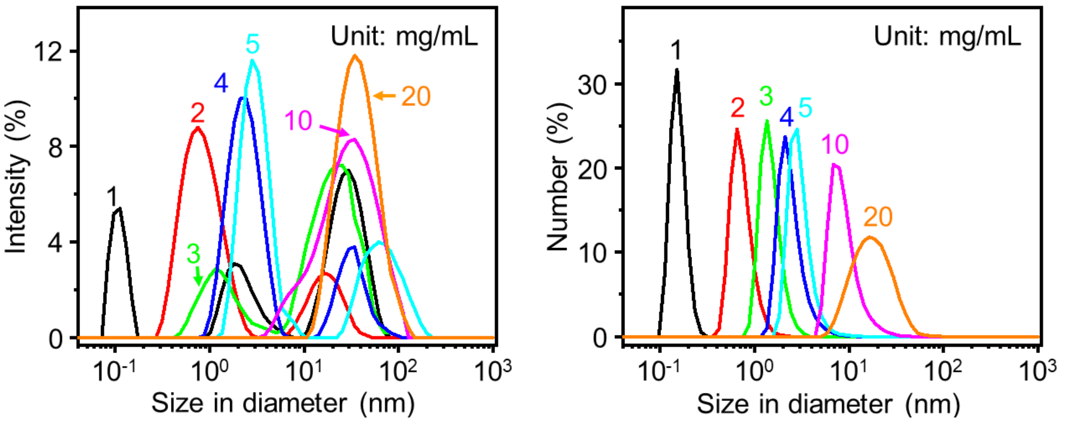

Figure S9 DLS data of Na-UPy-MnMo$_6$ aqueous solution with different concentration after incubation for 3 h.

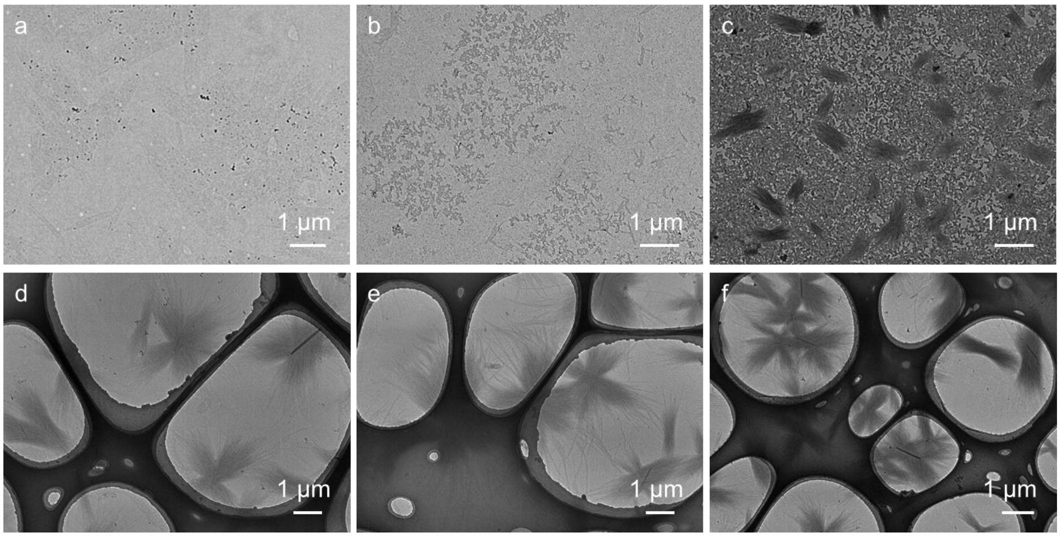

Figure S10 TEM images of Na-UPy-MnMo$_6$ self-assemblies in aqueous solution with different concentration after incubation for 3 h and air-drying for 1h, (a) 1 mg/mL, (b) 2 mg/mL, (c) 3 mg/mL, (d) 4 mg/mL, (e) 10 mg/mL, (f) 20 mg/mL.

S5. References

[1] Teunissen, A. J.; Paffen, T. F.; Ercolani, G.; De Greef, T. F.; Meijer, E. W. Regulating Competing Supramolecular Interactions Using Ligand Concentration. *J. Am. Chem. Soc.* **2016**, *138*, 6852−6860.

[2] Klemperer, W. G. Tetrabutylammonium isopolyoxometalates. *Inorg. Syn.* **1990**, *27*, 74–85.

[3] Marcoux, Pierre R.; Hasenknopf, B.; Vaissermann, J.; Gouzerh, P. Developing Remote Metal Binding Sites in Heteropolymolybdates. *Eur. J. Inorg. Chem.* **2003**, *2003*, 2406−2412.
